# Disease progression despite protective HLA expression in an HIV-infected transmission pair

**DOI:** 10.1186/s12977-015-0179-z

**Published:** 2015-06-30

**Authors:** Jacqui Brener, Astrid Gall, Rebecca Batorsky, Lynn Riddell, Soren Buus, Ellen Leitman, Paul Kellam, Todd Allen, Philip Goulder, Philippa C Matthews

**Affiliations:** Department of Paediatrics, Peter Medawar Building for Pathogen Research, University of Oxford, Oxford, OX1 3SY UK; Wellcome Trust Sanger Institute, Wellcome Trust Genome Campus, Hinxton, Cambridge, CB10 1SA UK; Ragon Institute of MGH, MIT and Harvard, Boston, MA USA; Integrated Sexual Health Services, Northamptonshire Healthcare NHS Foundation Trust, Northampton General Hospital, Cliftonville, Northampton, NN1 5BD UK; Department of International Health, Immunology and Microbiology, University of Copenhagen, Copenhagen N, Denmark; Division of Infection and Immunity, University College London, Gower Street, London, WC1E 6BT UK; Nuffield Department of Medicine, Peter Medawar Building for Pathogen Research, University of Oxford, Oxford, OX1 3SY UK

**Keywords:** HIV-1, HLA, CTL response, CRF01_AE Clade, Transmission pair, Ultra-deep sequencing

## Abstract

**Background:**

The precise immune responses mediated by HLA class I molecules such as HLA-B*27:05 and HLA-B*57:01 that protect against HIV disease progression remain unclear. We studied a CRF01_AE clade HIV infected donor-recipient transmission pair in which the recipient expressed both HLA-B*27:05 and HLA-B*57:01.

**Results:**

Within 4.5 years of diagnosis, the recipient had progressed to meet criteria for antiretroviral therapy initiation. We employed ultra-deep sequencing of the full-length virus genome in both donor and recipient as an unbiased approach by which to identify specific viral mutations selected in association with progression. Using a heat map method to highlight differences in the viral sequences between donor and recipient, we demonstrated that the majority of the recipient’s mutations outside of Env were within epitopes restricted by HLA-B*27:05 and HLA-B*57:01, including the well-studied Gag epitopes. The donor, who also expressed HLA alleles associated with disease protection, HLA-A*32:01/B*13:02/B*14:01, showed selection of mutations in parallel with disease progression within epitopes restricted by these protective alleles.

**Conclusions:**

These studies of full-length viral sequences in a transmission pair, both of whom expressed protective HLA alleles but nevertheless failed to control viremia, are consistent with previous reports pointing to the critical role of Gag-specific CD8+ T cell responses restricted by protective HLA molecules in maintaining immune control of HIV infection. The transmission of subtype CRF01_AE clade infection may have contributed to accelerated disease progression in this pair as a result of clade-specific sequence differences in immunodominant epitopes.

**Electronic supplementary material:**

The online version of this article (doi:10.1186/s12977-015-0179-z) contains supplementary material, which is available to authorized users.

## Background

Human leukocyte antigen (HLA) class I genotype has been consistently linked to outcome of HIV infection [1–5]. Among infected Caucasians, HLA-B*57 and HLA-B*27 are the best predictors of immune control [6, 7]. A better understanding of disease progression in subjects expressing protective HLA alleles such as these provides potentially valuable insights into the fundamental basis of HLA-mediated immune control, for which many distinct mechanisms have been proposed [8]. One mechanism believed to be important in contributing to the HLA associations with characteristic disease outcomes is the targeting of specific cytotoxic T lymphocyte (CTL) epitopes [9–17]. The subtype of HIV infection may therefore impact on disease control, by affecting the availability of certain specific T cell epitopes [18–20]. It remains unclear specifically which epitopes are most likely to induce the most effective anti-HIV immune responses. These considerations are important both for understanding the mechanisms of HLA-mediated immune control of viral replication and because CTL may play a critical role in HIV cure strategies [21].

Most studies of immune control in HIV-infected subjects expressing protective HLA alleles such as HLA-B*57:01 and B*27:05 have focused on Gag, and in particular the dominant CD8+ T cell responses targeting epitopes within p24 Gag. We investigated the case of an HIV infected transmission pair in which the recipient expressed both HLA-B*27:05 and HLA-57:01. Despite expression of these protective HLA alleles, disease progression occurred over four years from aviremia (viral load <50 copies/ml plasma) to antiretroviral therapy (ART) initiation, following a decline in absolute CD4 count to <350 cells/mm^3^. This study pursues the hypothesis that the CD8+ T cell epitopes important for immune control are those in which escape is selected in association with, or prior to, disease progression. Conversely, if escape has not occurred in parallel with disease progression, this would imply responses that do not protect against progression. We ultra-deep sequenced full-length HIV genomes using the Illumina MiSeq platform in both donor and recipient in order to define the mutations associated with disease progression.

## Results

### Progression in a UK transmission pair with CRF01_AE virus infection

We studied an adult Caucasian transmission pair from the UK. The male donor (HLA‐A*02:01/32:01 B*13:02/14:01 C*06:02/08:02) is believed to have acquired HIV infection in Thailand, and subsequently to have infected the female recipient (HLA-A*02:01/02:01 B*27:05/57:01 C*01:02/06:02) in the UK. Both partners were diagnosed more than 2 years later when the recipient was HIV-tested during pregnancy (referred to as ‘time 0’).

**Figure 1:**
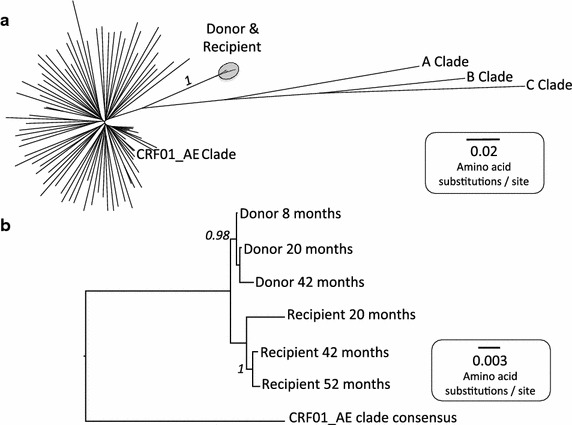
Phylogenetic trees demonstrating subtype and genetic proximity of sequences from an HIV transmission pair. **a** Maximum likelihood phylogenetic tree of 92 6803 bp nucleotide sequences, including consensus sequences for clades A, B, C and CRF01_AE, donor sequences from 8, 20 and 42 months post-diagnosis and recipient sequences from 20, 42 and 52 months post-diagnosis and 82 CRF01_AE Clade sequences from Thailand (Los Alamos HIV database, http://www.hiv.lanl.gov/). Donor and recipient sequences (circled) cluster with CRF01_AE clade sequences from Thailand, confirming that the Thai epidemic is the likely source of infection. The bootstrap value based on 1,000 bootstrap replicates for the donor-recipient cluster is shown in italics. **b** The close relationship between donor and recipient sequences supports transmission between these two subjects. Bootstrap values >0.75 based on 1,000 bootstrap replicates are shown in *italics*. *Scale bars* show number of substitutions per site.

**Figure 2:**
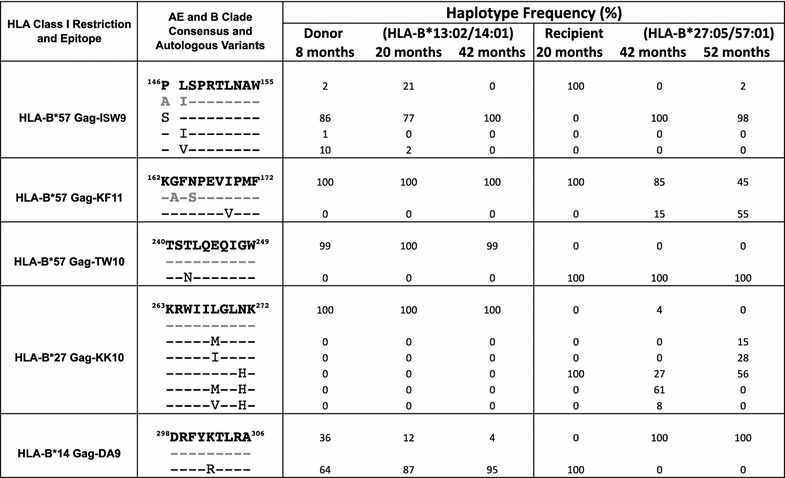
Sequence changes within Gag epitopes restricted by HLA-B*14, B*27 and B*57 identified by ultra-deep sequencing of an HIV transmission pair aligned to CRF01_AE clade consensus (*bold*) and B clade consensus sequence (*grey*). The frequency of minor variant haplotypes at the HLA-B*57 Gag-ISW9, HLA-B*57 Gag-KF11, HLA-B*57 Gag-TW10, HLA-B*27 Gag-KK10, and HLA-B*14 Gag-DA9 epitopes above a cut-off of 1% are shown. Depth of coverage ranges from 560 to 240,000 reads. Position 146 (flanking HLA-B*57 Gag-ISW9), associated with selection of an HLA-B*57/58:01-selected A146P processing mutation in B clade infection, is also shown. Minor variant frequencies are rounded off to the nearest 1%.

Using maximum likelihood analysis and Rega HIV-1 Subtyping Tool, we confirmed that the transmission pair was infected with CRF01_AE clade virus (Figure 1a and data not shown), the recombinant virus prevalent in Thailand [22, 23]. The close phylogenetic relationship of donor and recipient viruses suggests that these subjects are likely to be transmission partners (Figure 1b). As evidence to support the direction of transmission suggested by the clinical history, we found that an HLA-B*14:01 associated escape mutation, Gag-K302R (within the Gag-DA9 epitope [24]) present in the HLA-B*14:01-positive donor’s autologous virus, was transmitted to the HLA-B*14:01-negative recipient and subsequently reverted to wild-type in the recipient (Figure 2). In contrast, the HLA-B*27:05 and HLA-B*57:01-driven mutants observed in the recipient were not present in the donor.

**Figure 3:**
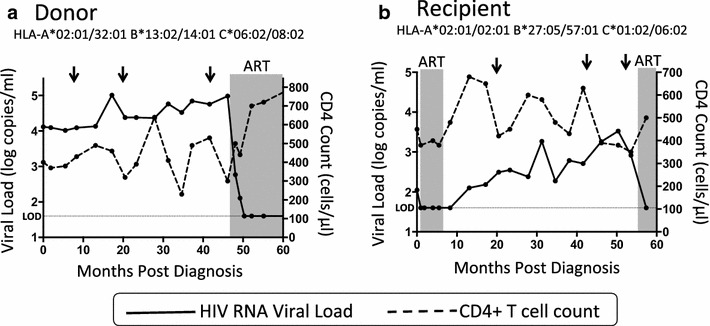
Course of infection in an HIV transmission pair. **a** Longitudinal plasma HIV RNA viral load and CD4+ T cell counts for the donor over 60 months of follow up. Grey shading represents the period during which the subject received antiretroviral therapy (ART) following decline of the CD4+ T cell count to <350cells/mm^3^ [58]. The horizontal dotted line represents the limit of detection (LOD) of the viral load assay (40 copies/ml). *Arrows* indicate the time of sampling for ultra-deep sequencing. **b** Longitudinal plasma HIV RNA viral load and CD4+ T cell counts for the recipient showing disease progression over 54 months of follow up. *Grey shading* represents the periods during which the subject received antiretroviral therapy (ART), initially as peri-partum prophylaxis and subsequently following decline of the CD4+ T cell count to 350cells/mm^3^ at 55 months post-diagnosis [58]. The *horizontal dotted line* represents the limit of detection (LOD) of the viral load assay (40 copies/ml). *Arrows* indicate the time of sampling for ultra-deep sequencing.

The HLA-B*27:05/57:01-positive recipient progressed to an absolute CD4+ T cell count of <350 cells/mm^3^, meeting the criteria for initiation of ART within 4.5 years of diagnosis (Figure 3b). The donor also progressed to ART initiation over a similar time period from diagnosis (Figure 3a) despite expressing three HLA molecules that have also been associated with some degree of protection against disease progression, HLA-A*32:01, HLA-B*13:02 and HLA-B*14:01 [6, 7, 25].

### HLA-B*27 and -B*57 Gag escape mutations in the recipient

**Figure 4:**
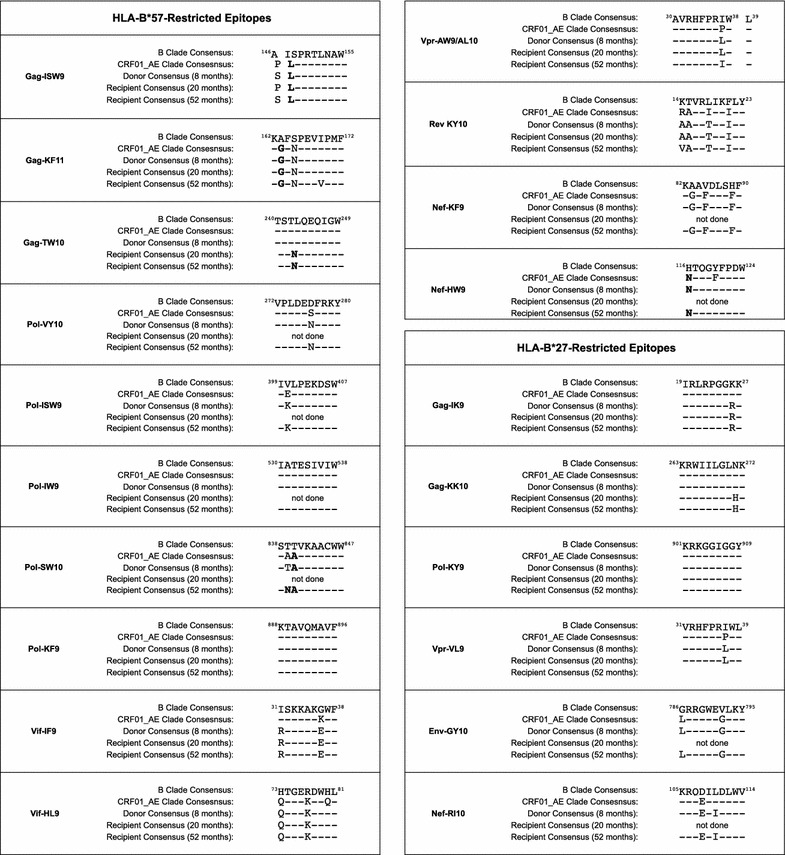
Alignment of HLA-B*27:05 and HLA-B*57:01-restricted epitopes in an HIV-1 transmission pair, showing sequences derived from donor and recipient, compared to consensus sequences for B clade and CRF01_AE clade. Known escape mutations in B clade infection are indicated in *bold*.

We initially focused on the well-studied Gag epitopes restricted by HLA-B*27:05 and HLA-B*57:01, believed to play a central role in immune containment in subjects expressing these alleles [8, 26–28]. The presence in the AE clade consensus of the very residues that are selected in B or C clade infected subjects as escape mutants in two of the four HLA-27:05/B*57:01-restricted p24 Gag-specific epitopes, ISPRTLNAW (Gag 147-155, ‘ISW9’) and KAFSPEVIPMF (Gag 162-172, ‘KF11’), prompted the question of whether well-defined HLA-B*27:05/57:01-restricted epitopes are accessible in AE clade infection. Only six out of 20 HLA-B*27/B*57-restricted epitopes (HLA-B*57 Gag-TW10, Pol-IW9, Pol-KF9; HLA-B*27 Gag-IK9, Gag-KK10, Pol-KY9) previously shown to drive the selection of escape mutants [24], share the same consensus sequence in B and AE clades (Figure 4).

In the HLA-B*27:05/57:01-positive recipient, the earliest sample was available for sequencing at 20 months following diagnosis, by which time progression was already evident (Figure 3b). The T242N mutation within the B*57:01-restricted epitope TSTLQEQIGW (Gag 240–249, ‘TW10’) had already reached fixation by this timepoint, being present in 100% of the intra-host population detected by ultra-deep sequencing (Figure 2). The other two HLA-B*57:01-restricted Gag epitopes, ISPRTLNAW (Gag 147–155, ‘ISW9’) and KAFSPEVIPMF (Gag 162–172, ‘KF11’) in consensus CRF01_AE Clade HIV already carry polymorphisms A146P/I147L and A163G/S165N that are well-characterized escape mutants within the B clade versions of these epitopes (Figure 4) [29, 30].

**Figure 5:**
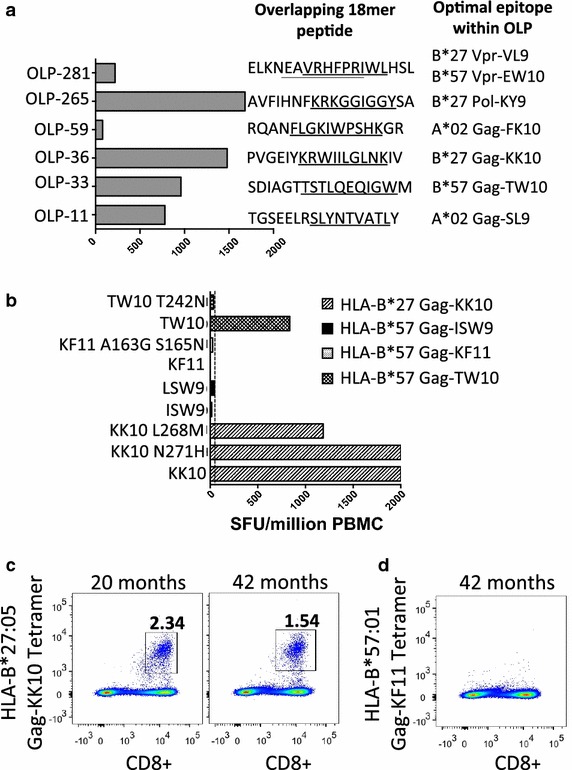
Quantification of CD8+ T cell responses to HLA-B*57 and HLA-B*27-restricted Gag epitopes in the recipient from an HIV transmission pair. **a** IFN-γ ELISpot responses at 20 months post-diagnosis in response to 410 18mer peptides spanning the B clade proteome. Responses to six 18mer peptides were detected. **b** IFN-γ ELISpot responses at 20 months post-diagnosis, showing maintenance of large responses to HLA-B*27 Gag-KK10 (KRWIIGLNK) and the N271H variant of this epitope and moderate responses to the HLA-B*57 Gag-TW10 epitope (TSTLQEQIGW), regardless of sequence changes within the autologous virus, with no responses to KF11 (KAFSPEVIPMF) or ISW9 (ISPRTLNAW) and their variants above the cut-off. Spot forming units (SFU) per million PBMC above a cut-off of 50 SFU/million are reported. Responses >2,000 SFU/million PBMC (OLP 36, KK10 and KK10-N271H) could not be quantified precisely. **c** HLA-B*27:05-KK10 tetramer stains at 20 and 42 months post-diagnosis show maintenance of a large Gag-KK10-specific CD8+ T cell population. **d** HLA-B*57:01-Gag-KF11 tetramer stain at 42 months post-diagnosis shows no detectable Gag-KF11-specific CD8+ T cell population.

To investigate which HIV-specific CD8+ T cell responses were detectable at the earliest timepoint available in the recipient (20 months post-diagnosis), we undertook IFN-γ elispot assays using a panel of 410 overlapping 18mer peptides spanning the HIV proteome [31], and identified responses to 6 of these 18mers (Figure 5a). The dominant Gag responses were to the HLA-B*27-restricted epitope KRWIILGLNK (Gag 263–272, ‘KK10’), and to the B*57:01-restricted epitope TSTLQEQIGW. In addition, there was a subdominant Vpr response and a high frequency response to the HLA-B*27:05-restricted epitope in Integrase, KRKGGIGGY (Pol 901-909, ‘KY9’), a response that is typically co-dominant with HLA-B*27:05 Gag-KK10 [32]. Whereas the HLA-B*27:05-KK10 and HLA-B*57:01-TW10 epitopes had escaped by the first timepoint (20 months in the recipient), this was not the case for the other epitopes. HLA-B*27:05 Pol-KY9 did not drive selection of escape even at 52 months post-diagnosis. These data support the hypothesis that the dominant Gag epitopes, including HLA-B*27:05-KK10 and the HLA-B*57:01-TW10, are critical for maintaining immune control.

Although an IFN-γ ELISpot response to the TW10 epitope was observed, no response to the autologous T242N variant was observed, and no CD8+ T cell responses were detectable in this subject to either the B clade or AE clade version of these ISW9 and KF11 epitopes (Figure 5). At 20 months after diagnosis, the HLA-B*27-restricted epitope KRWIILGLNK (Gag 263–272, ‘KK10’), believed to play an important role in HLA-B*27-mediated immune control of HIV [8, 26–28, 33], also contained the escape mutation N271H in 100% of the recipient sequences (Figure 2), despite persistence of a substantial ex vivo T cell response to the wild type and N271H variant epitopes (Figure 5b, c). Thus, in the case of the HLA-B*27:05/57:01-positive recipient, disease progression was seen in association with mutations in all four HLA-B27:05/*57:01-restricted p24 Gag epitopes.

### The majority of sequence changes selected in the recipient are escape polymorphisms in known epitopes

**Figure 6:**
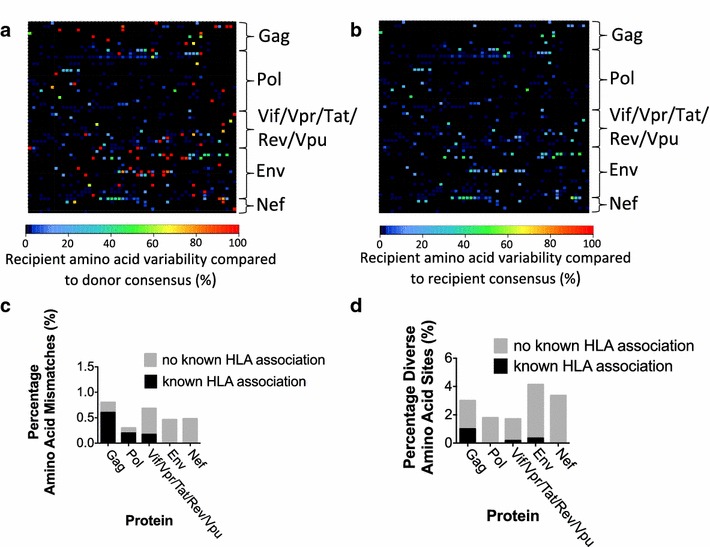
Comparison of inter- and intra-host diversity of HIV quasispecies in a transmission pair. **a** Heat map representation of inter-host amino acid diversity across the full-length HIV genome, comparing donor and recipient. For the baseline, we used the donor sequence at 8 months post-diagnosis (which in this case represents the closest approximation of the founder virus); variation in the recipient at 52 months post-diagnosis is compared to this baseline. Each *square* represents a single codon, coloured to reflect the percentage of sequences in the recipient that differ from the consensus residue in the donor. **b** Heat map representation of intra-host amino acid diversity in the recipient at 52 months post-diagnosis. For the baseline, we used the recipient consensus sequence at 52 months post-diagnosis to which the intra-host recipient population at the same timepoint is compared. Each *square* represents a single codon coloured to reflect the percentage of minor variants in the recipient that differ from the consensus (‘baseline’) residue. **c** Percentage of true amino acid mismatches (excluding positions where the recipient’s sequence was represented as a minor variant in the donor) between donor and recipient sequences by gene. The proportion of mismatches at sites where there is a known or predicted association with the recipient’s HLA alleles is indicated. **d** Percentage of diverse amino acid sites (variability >10%) in the recipient intra-host population by gene. The proportion of diverse sites where there is a known or predicted association with the recipient’s HLA alleles is indicated.

**Figure 7:**
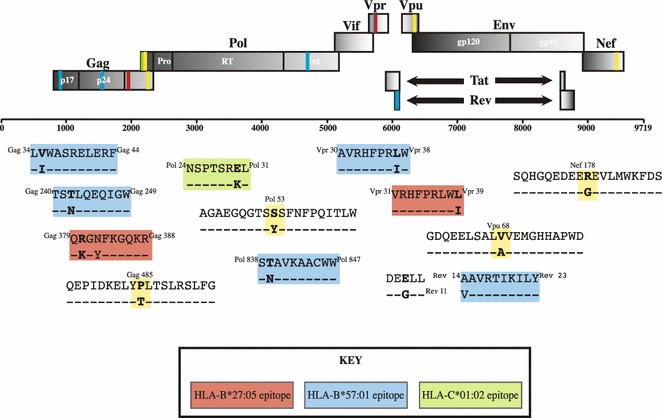
Schematic representation of sites of complete amino acid mismatch between the donor and recipient full-length HIV sequences. The recipient consensus sequence at 52 months is aligned to the donor consensus sequence at 8 months (the latter being the closest representation of the transmitted HIV sequence). The sites shown in this figure are complete mismatches between donor and recipient identified in the heat map analysis (*red squares*, Figure 6a) The mismatched residue is indicated in *bold*. HLA-B*27:05, B*57:01 and C*01:02-restricted epitopes are shown in *red*, *blue* and *green* respectively. Mismatched residues that do not fall within a relevant HLA-Class I epitope are shown in yellow.

To investigate whether other sequence changes outside of the well-studied region of p24 Gag might also have contributed to progression in the HLA-B*27:05/57:01-positive recipient, we next examined the ultra-deep sequence data of the full-length HIV genome. Heat maps were generated in order to visualize the proportion of amino acid variants at each position compared to a given baseline. We identified all sites of complete amino acid mismatch between the donor and recipient that reflect inter-host evolution using the donor consensus sequence at 8 months as the baseline for comparison to the recipient (Figure 6a). We also identified sites of amino acid diversity in the recipient at 52 months, demonstrating intra-host evolution, using the recipient consensus sequence at the same timepoint as the baseline for comparison (Figure 6b). The heat map analyses that were generated highlight the location of residues changing most rapidly in the recipient and which arose within CD8+ T cell epitopes (Figures 6, 7).

Using the donor consensus sequence at 8 months post-diagnosis as the closest approximation of the transmitted founder virus, we identified 16 residues across the full-length genome at which the residue in the donor (including minor variant residues) had been entirely replaced in the recipient by 52 months post-diagnosis. Excluding four residues within Env that are most likely to be susceptible to changes driven by neutralizing antibody responses, eight of the remaining 12 were in or flanking known epitopes, in seven cases either restricted by HLA-B*27:05 or HLA-B*57:01 (Figure 7). None of these sites are within epitopes restricted by HLA alleles expressed by the donor, indicating that these sequence changes are attributable to active selection in the recipient, rather than reversion of transmitted mutants selected in the donor.

Although these data from this single transmission pair do not definitively limit the most effective CD8+ T cell responses to this group of seven epitopes, these data are consistent with the hypothesis that the most effective responses are among this group. Of note, these do not include many of the well-studied HLA-B*27:05/57:01-restricted epitopes that, like the Gag epitopes, ISPRTLNAW (Gag 147–155, ‘ISW9’) and KAFSPEVIPMF (Gag 162–172, ‘KF11’), are mutated in AE clade compared to B clade virus (Figure 4), and in this transmission pair did not differ between donor and recipient at the timepoints compared.

To identify additional sites across the full-length genome in the recipient that were subject to turnover without having reached fixation yet, we sought sites at which amino acid diversity of at least 10% was present in the intra-host population at 52 months post diagnosis (Figure 6b, Additional file 1: Figure S1). This demonstrated diversity at only 2.6% of all amino acid residues, of which the majority (1.1%) were in Env, a highly variable region of the genome where mutations are driven largely by neutralizing antibody responses. Of the remaining sites of diversity, 16% were within or flanking recognized HLA-B*27/-B*57 epitopes. In both the donor/recipient comparison (Figure 6a) and intra-host diversity plot (Figure 6b) the evolving sites in Gag were frequently within known or predicted CTL epitopes restricted by the recipient’s HLA alleles, whereas those outside of Gag, especially in Env or Nef, were rarely within known or predicted epitopes (Figure 6c, d).

### Sequence changes in the donor reflect escape polymorphisms selected in known epitopes

Finally, we examined the sequences in the donor, who progressed despite possessing the protective HLA-A*32:01, HLA-B*13:02 and HLA-B*14:01 alleles. Compared to the full-length CRF01_AE clade consensus sequence, there are six epitopes at which HLA-associated mutations are present in the donor, two of which are in p24 Gag. These are within epitopes restricted by HLA-B*13:02 (Gag 135–143, ‘VV9’) and B*14:01 (Gag 298–306, ‘DA9’) respectively (Additional file 2: Figure S2). Thus, as in the recipient, progression to HIV disease in the donor was associated with mutations in critical p24 Gag epitopes.

## Discussion

This study capitalizes on longitudinal data from a well-characterized transmission pair, for whom we were able to maximize the depth (ultra-deep approach) and breadth (full-length HIV genomes) of sequence resolution. This allowed us to quantify precisely the evolution of escape mutations, including minor variants, in the context of what would usually be regarded as a highly favorable combination of HLA alleles, HLA-B*27:05 and HLA-B*57:01. Since both these alleles occur at a very low frequency within the Thai population (approximately 0.2 and 1.4% respectively [34]) finding this haplotype in the context of CRF01_AE clade infection is an ‘accident of nature’ which provides a unique opportunity to study the mechanisms of immune control.

There are conflicting data regarding the extent to which HLA-B*57 may be protective in Thai cohorts. Although a recent study in a particular Thai cohort, where the median CD4+ T cell count was only 86 T cells/mm^3^, reported that HLA-B*57:01 was protective [34], a parallel study of 116 transmission pairs found no benefit of HLA-B*57 [35]. The latter result fits with the picture we describe in our HLA-B*57-positive recipient, and is consistent with the abrogation of HLA-B*57-restricted Gag epitopes due to pre-existing polymorphisms in CRF01_AE clade virus that represent HLA-B*57 escape mutations. This highlights the extent to which clade of infection may be an important determinant of immunological and clinical outcomes; even in an individual expressing a favourable combination of HLA alleles that are usually strongly linked to immune control, rapid progression may result in the context of infection with a viral sequence bearing pre-existing escape mutations.

Although HIV is recognised as a highly polymorphic virus, this study demonstrates that viral evolution is frequently constrained to specific amino acid residues, with the success of the CD8+ T cell response dependent on these sites. In fact, significant variability (>10%) was evident within the intra-host population at only 2.6% of amino acid residues in the recipient. Ultra-deep sequencing demonstrated a high degree of conservation within key HLA-B*27:05 and HLA-B*57:01-restricted epitopes (Figure 2), with the exceptions being at pre-defined sites of escape mutation, most often corresponding to anchor residues. This points to selection pressure that is very specifically directed at these particular sites, consistent with previous reports showing that selective escape from CD8+ T cell responses follows constrained evolutionary pathways [36].

Consistent with previous reports [35, 37], in the recipient, we observed the robust selection of the Gag HLA-B*57-selected T242N mutation in the Gag-TW10 epitope, that reaches fixation and is maintained in the host viral population. Within the Gag-KK10 epitope, strong selection pressure drives N271H selection almost to fixation. Subsequent reversion to the wildtype residue in a substantial proportion of the variants does, however, indicate more complexity in the adaption of the autologous virus at this site.

Explaining variation at certain sites is made more complicated by multiple influences on viral polymorphism. For example, Gag P146S is a common variant in CRF01_AE Clade infection (occurring in approximately 9.5% of sequences), but this site is also subject to selection pressure from both HLA-B*13:02 and HLA-B*57:01-mediated T cell responses [12, 18, 24]. Variation at this position in our study could therefore be attributed to selection pressure from either the donor or recipient CD8+ T cell response, or to a founder virus bearing a serine variant rather than the more common proline. An alternative explanation for sequence variation occurring over time in a transmission pair is that more than one transmission event has taken place; the introduction of a new founder virus could then alter the dominant quasispecies. In this instance, re-infection appears unlikely on the basis of phylogeny demonstrating clear clustering of donor and recipient sequences respectively, but cannot be excluded completely due to the limited number of samples analyzed over the time period of follow up.

It is striking that even by applying an unbiased approach to seeking sequence variability across the whole genome, the majority of polymorphisms identified in the recipient were within or flanking known epitopes, with HLA-B*27 and HLA-B*57-restricted epitopes being dominant, and Gag accounting for the greatest number of these. The observations made here, using the approach of this genome-wide search for polymorphisms, therefore corroborate previous data in studies that have used known CD8+ T cell epitopes or IFN-γ ELISpot assays as their starting point to identify sites of immune selection [30, 32, 37].

The unique nature of the circumstances described in this report mean that the findings are difficult to replicate, and can be presented as a case study only. An additional limitation for this transmission pair was lack of information about the precise timing of infection, and absence of samples from timepoints closer to the time of transmission. Furthermore, a lack of data on the epitopes restricted in the context of this rare combination of HLA allele and clade of infection has limited our analysis of epitopes to those that have been described in the context of B clade infection. It is noteworthy, for example, that the B*27:05-KK10 variant selected in the clade AE-infected recipient was N271H that has been rarely observed in B clade infection. In this case, a strong N271H-specific CTL response was observed, which may appear counter-intuitive if N271H is selected as an escape mutant. However, it has been well described with respect to escape mutants that affect T cell receptor recognition, such as the more commonly observed L268M within KK10 [38–40], that a high frequency response can be observed to a TCR-variant when it is recognised by a subset of CTL clones. Despite these caveats, this transmission pair provided a unique insight, gained by full-length ultra-deep sequencing data, supporting the association between the selection of polymorphisms to allow escape from HLA-B*27 and HLA-B*57-restricted epitopes, and loss of immunological control.

## Conclusions

The unique opportunity to study CRF01_AE Clade HIV infection longitudinally in the context of a transmission pair with protective HLA alleles, using ultra-deep sequencing and an unbiased approach to full-length sequence analysis, has shown the extent to which the polymorphisms associated with disease progression are constrained to very specific amino acid sites, frequently within Gag-restricted epitopes. The extent to which selection of escape mutations is robust and predictable is surprising given the overall plasticity of the HIV genome. This observation is encouraging for the development of T cell vaccines for which meeting the challenges presented by viral escape is a major consideration.

## Methods

### Study subjects

This adult Caucasian transmission pair was recruited from the Thames Valley Cohort, UK, previously described [32]. A male donor, infected prior to 2007 subsequently infected his female partner. Both subjects gave written informed consent for their participation. Ethics approval was given by the Oxford Research Ethics Committee.

### HLA typing

DNA extraction was performed from whole blood using PureGene reagents (Qiagen, UK). Four-digit high resolution Sequence Based Typing of HLA-A, -B, and -C was performed from genomic DNA in the CLIA/ASHI accredited laboratory of William Hildebrand, PhD, (ABHI) at the University of Oklahoma Health Sciences Center using a locus specific PCR amplification strategy and a heterozygous DNA sequencing methodology for exon 2 and 3 of the class I PCR amplicon. Relevant ambiguities [41] were resolved by homozygous sequencing.

### Viral load and CD4 testing

HIV viral load testing was performed using the Roche Amplicor version 1.5 assay (Roche, Switzerland). CD4+ T cell counts were determined by flow cytometry.

### RNA extractions and viral amplification using PCR

RNA extractions were performed using the Qiamp Viral RNA Mini Kit (Qiagen, UK). 1 ml aliquots of plasma were centrifuged for 1 h at 21,000 rpm and 860 μl of supernatant removed before proceeding according to the manufacturer’s instructions. Samples with a viral load below 3,000 copies/ml were concentrated by processing 3 aliquots of plasma on the same Qiamp column. PCR amplification of the full HIV genome was performed in four fragments using Superscript III One-Step RT PCR Kit with Platinum Taq High Fidelity enzyme (Invitrogen, UK) as previously described [42].

### Ultra-deep sequencing and de novo assembly of consensus sequences

Ultra-deep sequencing of the HIV genome (complete amino acid coding region and partial long terminal repeats) was performed as previously described [43]. Amplicons were pooled for Illumina library preparation, including a unique bar code for each sample, and sequenced using MiSeq 250 bp paired-end technology in a pool of 9, 15 and 27 libraries, respectively [44]. Quality control (removing reads of <50 bp and trimming low-quality bases from the 3′-end of the reads until the median quality of the read was 30) was carried out using QUASR (http://www.sourceforge.net/projects/quasr/). A de novo assembly was constructed using SPAdes version 2.4.0 [45]. Resulting contiguous sequences were aligned with the sequence of the HIV CRF01_AE reference strain CM240 (accession number U54771), and a consensus sequence was generated using Abacas version 1.3.1 and MUMmer version 3.2 [46].

### Minor variant analysis

The raw reads were assembled by *Vicuna* [47] and *V*-*FAT* [48] to form a single genome, which represents the majority base at each nucleotide position (the consensus assembly). The reads were then aligned to the consensus assembly using *Mosaik* [49]. *V*-*Phaser*2 [50] was used in order to call variants. This program uses both quality scores as well as covariation between variants (observation of two variants on the same read) to separate real variants from sequencing artifacts. We applied a modified strand bias cut-off to the variant calls. We required the odds-ratio of the appearance of a mutation between the two directions to be larger than 3.

### Heat map analysis

Heat maps of intra-host diversity were created using *Vprofiler* [51] as well as custom programs written in Perl and R. We carried out diversity heat map analysis on the recipient at 52 months post-diagnosis (the earliest timepoint at which full-length sequencing data were available) and on the donor at 8 months post-diagnosis (representing the timepoint closest to the time of transmission). This method provides colour plots that represent the extent of variability across the HIV proteome, either comparing sequences between two individuals (in this case, donor and recipient), or representing diversity within one individual at a given time point (in this case, providing a snapshot of within-host diversity in the recipient at time 52 months).

### Determination of haplotypes

Haplotypes in the epitope regions were determined using *Vprofiler* [51] by selecting reads that span the epitope region and which contain only accepted variants. This analysis is limited to positions that are within the sequence read length of 250 bp.

### Epitopes known or predicted to be restricted by expressed HLA-alleles

We focused on HLA-B restricted epitopes, since the HLA-B alleles are most strongly linked to HIV disease outcome in HIV infection [52] and there are no significant HLA associations with disease control for the HLA-A and HLA-C alleles expressed by this transmission pair. Known epitopes were identified from The Los Alamos Immunology Database CTL Epitopes A-list [53]. Predicted epitopes were identified from described HLA footprints [24] and the *HLArestrictor* tool [54].

### IFN-γ ELISpot assays

We tested cryopreserved peripheral blood mononuclear cells (PBMC) from the recipient collected at 20 and 42 months post-diagnosis against HLA-B*27:05 and HLA-B*57-restricted Gag epitope peptides including CRF01_AE clade-specific peptide variants, to screen for IFN-γ ELISpot responses as previously described [31]. IFN-γ ELISpot responses to 410 18mer overlapping peptides spanning the B clade proteome were also tested, as previously described [31].

### Phylogenetic analysis

Maximum likelihood phylogenetic trees using the general time reversible model of nucleotide substitution, as determined by jModelTest version 0.1.1 [55], were constructed from near-full genome (6,803 bp) data with 1,000 bootstrap replicates using Mega 6.06 software and viewed using FigTree v1.4.0 software. Clade consensus sequences were generated using full-length sequences and the Simple Consensus Maker tool available from the Los Alamos HIV database (http://www.hiv.lanl.gov/). Eighty-two full-length AE clade reference sequences from Thailand collected from the same database were included as reference sequences. All reference sequences were based on data collected after 2004. Sequence subtypes were confirmed using REGA HIV-1 Subtyping Tool version 3.0 [56].

### Peptide-MHC tetramer staining and flow cytometry

Peptide-MHC tetramers were generated as previously described [57]. Cryopreserved PBMC (1 million per stain) from the recipient collected at 20 and 42 months post-diagnosis were stained with PE-conjugated HLA-B*27:05-KK10 and HLA-B*57:01-KF11 peptide-MHC tetramers, anti-CD3 Pacific Orange (Invitrogen, UK), anti-CD4 AlexaFlour700 (BD Biosciences, UK) and ant-CD8 V450 (BD Biosciences, UK) antibodies and near-IR Live/Dead marker (Invitrogen, UK). Samples were analyzed using an LSRII flow cytometer (BD, UK) collecting a minimum of 500,000 events and gating on singlets, lymphocytes, live cells and CD3 + cells. Data were analyzed using FlowJo version 10.0.7.

### Sequence accession numbers

The Illumina MiSeq sequencing data obtained in this study are available from the EMBL/GenBank/DDBJ Sequence Read Archive under accession numbers: ERS250039, ERS250040, ERS250041, ERS250042, ERS394610 and ERS394611. Consensus sequences have been deposited in GenBank under the accession numbers: KP873161-KP873166.

## Additional files

**Additional file 1: Figure S1.** Sites of amino acid diversity in the recipient from an HIV transmission pair at 52 months post-diagnosis. Diverse sites are defined as amino acid positions with diversity of ≥10% in the intra-host population. Sites that fall within or flanking known or predicted HLA-B*27:05, B*57:01 and C*01:02-restricted epitopes are shown in red, blue and green respectively. Duplicated sites, present due to overlap in the Gag/Pol and Vif/Vpr reading frames, are shown in grey.
**Additional file 2: Figure S2.** Sites of HLA-associated footprints in the donor from an HIV transmission pair at 8 months post-diagnosis aligned to B and CRF01_AE clade consensus sequence. Epitopes restricted by the favourable alleles expressed by the donor, A*3201, B*1302 and B*1401, are shown.

## Abbreviations

ART: Antiretroviral therapy
CTL: cytotoxic T lymphocyte
DNA: deoxyribonucleic acid
ELISpot: enzyme-linked Immunosorbent spot assay
Env: envelope glycoproteins
Gag: group-specific antigen (capsid protein)
HIV: human immunodeficiency virus
HLA: human leukocyte antigen
IFN: interferon
MHC: major histocompatibility complex
Nef: negative regulatory Factor protein
PBMC: peripheral blood mononuclear cells
PCR: polymerase chain reaction
PE: phycoerythrin
Pol: protease, reverse transcriptase and integrase polyprotein
Rev: regulator of expression of virion proteins
RNA: ribonucleic acid
RT: reverse transcriptase
Tat: trans-activator of HIV-1 gene expression
Vif: viral infectivity factor
Vpr: viral protein R
Vpu: viral protein U.
