# Supplementary figures and images for: Disease progression despite protective HLA expression in an HIV-infected transmission pair
Source: Retrovirology. 2015 Jun 30;12:55. doi: 10.1186/s12977-015-0179-z (PMC4487201; doi:10.1186/s12977-015-0179-z)

Figure S1

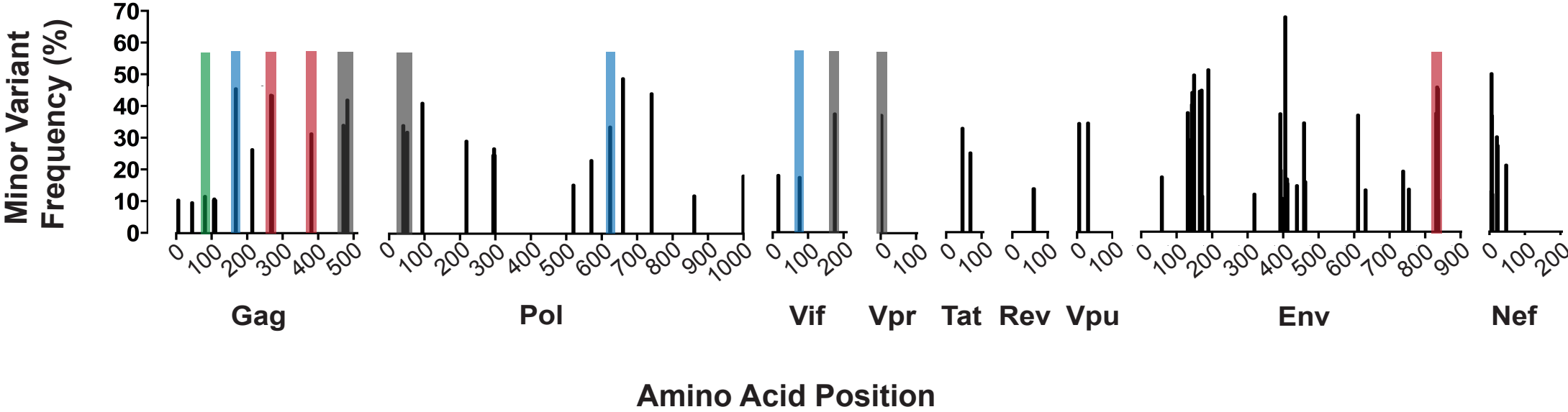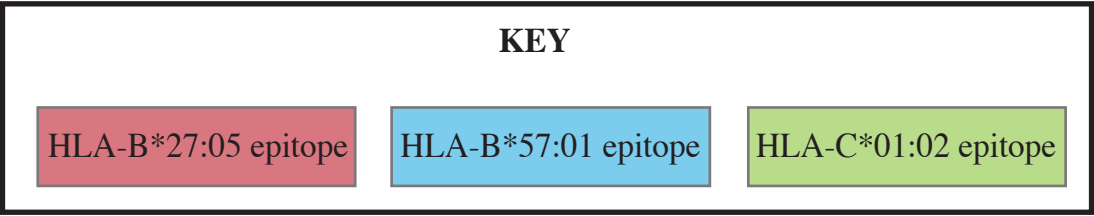

Supplement: Supplementary file 1 — Additional file 1: Figure S1. Sites of amino acid diversity in the recipient from an HIV transmission pair at 52 months post-diagnosis. Diverse sites are defined as amino acid positions with diversity of ≥10% in the intra-host population. Sites that fall within or flanking known or predicted HLA-B*27:05, B*57:01 and C*01:02-restricted epitopes are shown in red, blue and green respectively. Duplicated sites, present due to overlap in the Gag/Pol and Vif/Vpr reading frames, are shown in grey. [file 12977_2015_179_MOESM1_ESM.pdf]
