# Supplementary material for: Disease progression despite protective HLA expression in an HIV-infected transmission pair
Source: Retrovirology. 2015 Jun 30;12:55. doi: 10.1186/s12977-015-0179-z (PMC4487201; doi:10.1186/s12977-015-0179-z)
Supplement: Supplementary file 2 — Additional file 2: Figure S2. Sites of HLA-associated footprints in the donor from an HIV transmission pair at 8 months post-diagnosis aligned to B and CRF01_AE clade consensus sequence. Epitopes restricted by the favourable alleles expressed by the donor, A*3201, B*1302 and B*1401, are shown. [file 12977_2015_179_MOESM2_ESM.pdf]

Figure S2: Sites of HLA-associated footprints in the donor from an HIV transmission pair at 8 months post-diagnosis aligned to B and CRF01_AE clade consensus sequence. Epitopes restricted by the favourable alleles expressed by the donor, A*3201, B*1302 and B*1401, are shown.

HLA-A*32:01-Restricted Epitopes

Epitope		
Pol-PW10	B Clade Consensus:	^{547}PIQKETWETW^{556}
	Described B Clade Footprint:	---R----I-
	CRF01_AE Clade Consesnsus:	---R------
	Donor Consensus (8 months):	---R------
Pol-KF9	B Clade Consensus:	^{930}KQITKIQNF^{938}
	Described B Clade Footprint:	---I-M---
	CRF01_AE Clade Consesnsus:	---------
	Donor Consensus (8 months):	---------
Vif-KY11	B Clade Consensus:	^{34}KAKGWFYRHHY^{44}
	Described B Clade Footprint:	-------K---
	CRF01_AE Clade Consesnsus:	---K-------
	Donor Consensus (8 months):	---E-------
Tat-KI11	B Clade Consensus:	^{29}KCCFHCQVCFI^{39}
	Described B Clade Footprint:	Q----------
	CRF01_AE Clade Consesnsus:	---Y------L
	Donor Consensus (8 months):	---W---L--L
Env-RW9	B Clade Consensus:	^{419}RIKQIIN**M**W^{427}
	Described B Clade Footprint:	-------**L**-
	CRF01_AE Clade Consesnsus:	K--------
	Donor Consensus (8 months):	K-R--VR--
Env-SY10	B Clade Consensus:	^{703}SIVNRNRQGY^{712}
	Described B Clade Footprint:	-LA-------
	CRF01_AE Clade Consesnsus:	-----V----
	Donor Consensus (8 months):	--A--V----

HLA-B*14:01-Restricted Epitopes

Epitope		
Gag-DA9	B Clade Consensus:	^{298}DRFYKTLRA^{306}
	Described B Clade Footprint:	----R----
	CRF01_AE Clade Consesnsus:	---------
	Donor Consensus (8 months):	----R----
Vif-DK8	B Clade Consensus:	^{172}DRWNKPQK^{179}
	Described B Clade Footprint:	----E-K-
	CRF01_AE Clade Consesnsus:	--------
	Donor Consensus (8 months):	-K------
Env-EL9	B Clade Consensus:	^{584}ERYLKDQQL^{592}
	Described B Clade Footprint:	-XF-Q---F
	CRF01_AE Clade Consesnsus:	-------KF
	Donor Consensus (8 months):	-------KF

HLA-B*13:02-Restricted Epitopes

Epitope		
Gag-VV9	B Clade Consensus:	^{135}VQNLQGQMV^{14} HQAI^{147}
	Described B Clade Footprint:	--------- --SL
	CRF01_AE Clade Consesnsus:	---A----- ---L
	Donor Consensus (8 months):	---A----- --SL
Gag-RI9	B Clade Consensus:	^{429}RQANFLGKI^{437}
	Described B Clade Footprint:	-------RL
	CRF01_AE Clade Consesnsus:	---------
	Donor Consensus (8 months):	---------
Pol-RI10	B Clade Consensus:	^{113}RQYDQILIEI^{122}
	Described B Clade Footprint:	------X---
	CRF01_AE Clade Consesnsus:	----------
	Donor Consensus (8 months):	------C---
Pol-KI10-	B Clade Consensus:	^{521}KQLTEAVQKI^{530}
	Described B Clade Footprint:	---V------
	CRF01_AE Clade Consesnsus:	----------
	Donor Consensus (8 months):	----------
Nef-RV9	B Clade Consensus:	^{106}RQDILDLWV^{114}
	Described B Clade Footprint:	-R-------
	CRF01_AE Clade Consesnsus:	--E------
	Donor Consensus (8 months):	-RE-I----

HLA-Associated Footprints Outside Epitopes

Footprint		
HLA-A*32:01 Vpu	B Clade Consensus:	^{80}D
	Described B Clade Footprint:	V
	CRF01_AE Clade Consesnsus:	-
	Donor Consensus (8 months):	-
HLA-A*32:01 Env	B Clade Consensus:	^{121}KLTPLCVTLNCTDLM^{135}
	Described B Clade Footprint:	--------------R
	CRF01_AE Clade Consesnsus:	------------NAN
	Donor Consensus (8 months):	---------H-KKVD
HLA-B*14:01 Vpr	B Clade Consensus:	^{3}Q
	Described B Clade Footprint:	R
	CRF01_AE Clade Consesnsus:	-
	Donor Consensus (8 months):	-
B*14 Nef	B Clade Consensus:	^{53}A
	Described B Clade Footprint:	P
	CRF01_AE Clade Consesnsus:	-
	Donor Consensus (8 months):	-
B*14 Nef	B Clade Consensus:	^{101}I
	Described B Clade Footprint:	V
	CRF01_AE Clade Consesnsus:	-
	Donor Consensus (8 months):	-
